# Integration of whole genome resequencing and transcriptome sequencing to identify candidate genes for tall and short traits in Baicheng Fatty chickens

**DOI:** 10.3389/fvets.2025.1534742

**Published:** 2025-02-27

**Authors:** Jiaqi Li, Kaixu Chen, Mengting Zhu, Jingdong Bi, Honggang Tang, Weiyi Gao

**Affiliations:** College of Animal Science, Xinjiang Agricultural University, Urumqi, China

**Keywords:** Baicheng Fatty chicken, whole genome resequencing, transcriptome sequencing, tibia length, *KLF15*

## Abstract

The tall and short traits of chickens are significant indicators for evaluating their growth and development. Tall chickens have longer growth cycles, allowing them to accumulate sufficient nutrients and resulting in superior meat quality. This study aims to investigate the tall and short traits of Baicheng Fatty chickens and to identify relevant candidate genes. A total of 25 Baicheng Fatty chickens were selected for this research, where whole genome resequencing was performed on all samples to uncover genetic variations influencing tall and short traits. Additionally, transcriptome sequencing was conducted on 15 of these chickens to identify important genes affecting these traits through combined analysis. Using methods such as population genetic structure analysis, principal component analysis (PCA), linkage disequilibrium analysis (LD), runs of homozygosity (ROH) analysis, as well as genetic differentiation index (*F_ST_*) and nucleotide diversity (θπ), a total of 1,019 candidate genes were identified through whole genome resequencing analysis. Gene Ontology (GO) and Kyoto Encyclopedia of Genes and Genomes (KEGG) enrichment analyses were performed on these candidates. From the transcriptome data, 253 differentially expressed genes (DEGs) were identified, including 229 upregulated and 24 downregulated genes. GO and KEGG enrichment analyses were conducted on these differential genes, and a protein–protein interaction network for the DEGs was constructed. Through the combined analysis of whole genome resequencing and transcriptome data, six intersecting genes were identified: *KLF15*, *NRXN1*, *LOC107050638*, *MHCY11*, *HAO1*, and *BORCS6*. KEGG enrichment analysis revealed significant involvement in the Glyoxylate and Dicarboxylate Metabolism pathway, Peroxisome pathway, Carbon Metabolism, and Cell Adhesion Molecules (CAMs) pathway. These genes may influence the growth and developmental patterns of skeletal structures, though their regulatory mechanisms require further investigation. This study provides new insights for further research into the genetic mechanisms underlying chicken skeletal development and growth, as well as potential molecular markers for poultry breeding.

## 1 Introduction

The Baicheng Fatty chicken is a local breed from the Aksu region of Xinjiang, known for its excellent meat quality, cold resistance, tolerance to rough feeding, and strong disease resistance (1–4). Based on their leg type, Baicheng Fatty chickens can be divided into two types: high-legged and short-legged. The high-legged Baicheng Fatty chicken has a larger body size, longer legs, and is adapted to complex terrain, demonstrating strong mobility and foraging abilities (5). The short-legged Baicheng Fatty chicken, on the other hand, has shorter legs and a compact body, typically with higher meat quality and better feed conversion rates.

Current research primarily focuses on the overall production performance and genetic diversity of Baicheng Fatty chickens (6–10), but the genetic mechanisms of leg traits remain underexplored, especially the genetic basis of high and short leg traits. Studies show that skeletal development plays a crucial role in the growth of poultry, particularly the development of leg bones, which directly impacts the mobility and production performance of poultry (11–14). Bone marrow, as a key site for skeletal development, is not only a hematopoietic tissue but also the source of mesenchymal stem cells required for bone growth. Mesenchymal stem cells in bone marrow can differentiate into osteoblasts, playing a crucial role in bone formation and remodeling (15).

Currently, research on the genetic mechanisms of leg traits in chickens is scarce, particularly regarding the genetic control of high and short legs in Baicheng Fatty chickens, which remains unclear (16–18). Wang et al. (6) conducted a systematic study on the dwarf gene in Xingyi short-legged chickens, finding that the traits of short and high legs are controlled by a pair of alleles on an autosome, with short legs being dominant over high legs. However, this study was limited to the role of a single gene and did not reveal the molecular mechanisms at the regulatory network level. Recent studies have shown that the expression levels of growth factors such as GH and IGF-1, and their receptor genes, are closely related to skeletal development in chickens (19). Moreover, transcriptomic research indicates that genes from families such as collagen type COL and BMP play important roles in skeletal development (20).

Despite this, there are still the following limitations in the systematic research on high and short leg traits in Baicheng Fatty chickens: (a) a lack of variation site screening at the whole-genome level; (b) an inability to clarify the key gene expression patterns related to skeletal development in bone marrow tissue; (c) a lack of in-depth analysis of the regulatory networks. This study integrates whole-genome resequencing data and bone marrow transcriptome data to systematically screen candidate genes related to the high and short leg traits of Baicheng Fatty chickens from both genomic and transcriptomic perspectives. The goal is to reveal the molecular regulatory network of this trait, providing scientific evidence for the conservation and genetic improvement of the Baicheng Fatty chicken breed.

## 2 Materials and methods

### 2.1 Experimental animals and sample preparation

The experiment was conducted under uniform rearing conditions, with standardized feeding and immunization protocols, and free access to water. The tall-legged and short-legged traits of Baicheng Fatty chickens were observed. A total of 25 Baicheng Fatty chickens, aged 18 months (12 with tall legs and 13 with short legs), bred by Xinjiang Jinyou Native Animal Husbandry Co., Ltd., were selected for the study. Eighteen months was chosen because by this age, the chickens’ bones are fully developed, making it an ideal time to study skeletal maintenance and potential degenerative changes. At this stage, the bones are stable but have not yet begun to deteriorate, providing a key window to examine how genetic and environmental factors influence leg traits. In accordance with the guidelines of the Institutional Animal Care and Use Committee of Xinjiang Agricultural University (Approval No.: 2021099), bone marrow samples were collected from the femurs of all 25 chickens. The chickens were used for whole genome resequencing (all 25), with 15 selected for transcriptome sequencing. Of the 15 chickens chosen for transcriptome sequencing, 7 were males (4 with tall legs and 3 with short legs) and 8 were females (4 with tall legs and 4 with short legs). The samples were quickly placed in liquid nitrogen and stored at −80°C for future use. The experiment took place in November 2023 at the experimental base of Xinjiang Agricultural University.

### 2.2 Whole genome resequencing

#### 2.2.1 DNA extraction and basic data processing

Genomic DNA was extracted from the 25 samples using the phenol-chloroform method. The concentration and purity of the DNA samples were assessed using a NanoDrop 2000 microvolume UV spectrophotometer. Gel electrophoresis was employed to evaluate the integrity and degradation of the DNA samples, providing a comprehensive assessment of DNA quality. After passing the quality inspection, the samples were sent to Beijing Baimake Biotechnology Co., Ltd. for library construction and paired-end sequencing. To ensure the accuracy of subsequent analyses, the raw sequencing data were quality-controlled using Fastp software. The filtered clean data were aligned to the chicken reference genome (Gallus_gallus.GRCg7b.genome.fa) using BWA (11), sorted with the sort command in Samtools, and duplicates were removed with Picard. Subsequently, statistical analysis of the sorted BAM files was performed using Qualimap software (12). The Genome Analysis Toolkit (GATK) was utilized for whole genome variant detection. The filtering criteria for population SNPs included: exclusion of SNPs with a minor allele frequency below 5%, removal of loci with a missing rate greater than 10%, and exclusion of SNPs with a *p*-value less than 1e-5 in Hardy–Weinberg equilibrium tests. SNPs were annotated using ANNOVAR software.

#### 2.2.2 Population genetic analysis

##### 2.2.2.1 Principal component analysis (PCA)

VCF files were converted to PLINK format using VCFtools v0.1.17, and PCA was performed using PLINK v1.90 (13). The results were visualized with the R package ggplot2.

##### 2.2.2.2 Linkage disequilibrium (LD)

LD at varying distances was assessed by calculating *r*^2^ values using software such as PLINK, with the LD coefficient represented by *r*^2^ and the distance corresponding to half of the maximum *r*^2^ value defined as the LD decay distance (14).

##### 2.2.2.3 Runs of homozygosity (ROH) analysis

Genotype data were quality-controlled using PLINK to remove low-quality variants. A fixed-size sliding window was applied to scan the chromosomes for contiguous homozygous SNPs. ROH segments were identified and recorded based on predefined thresholds. Specifically, the following parameters were used for ROH detection: the minimum length of ROH segments was 100 kb, each ROH was required to include at least 10 SNPs, the minimum density of homozygous SNPs within a segment was set to 10, each sliding window contained at least 50 SNPs, a maximum of 1 heterozygous SNP was allowed per window, and the maximum gap between homozygous SNPs within the window was 100 SNPs.

##### 2.2.2.4 Joint analysis of genetic differentiation index (*F_ST_*) and nucleotide diversity (θπ)

The *F_ST_* value reflects the degree of population differentiation, with values ranging from 0 to 1. When *F_ST_* is between 0 and 0.05, it indicates low genetic differentiation between populations. A value between 0.05 and 0.15 suggests moderate differentiation, while values from 0.15 to 0.25 indicate a relatively high level of differentiation. *F_ST_* values greater than 0.25 are considered to represent substantial genetic differentiation between populations (15). In this study, *F_ST_* values for each sliding window were calculated using VCFtools with a window size of 40 kb and a step size of 20 kb. The intersection of the top 1% of *F_ST_* results and the top 1% of *π* ratio results was taken as the selected regions.

##### 2.2.2.5 GO enrichment and KEGG pathway analysis of genes in selected regions

Gene Ontology (GO) and Kyoto Encyclopedia of Genes and Genomes (KEGG) enrichment analyses were conducted using DAVID.^1^ Subsequently, the enrichment results were visualized using the online plotting software WeiShengXin.^2^

### 2.3 Transcriptome sequencing

#### 2.3.1 RNA extraction and library construction

Total RNA was extracted from 15 chicken bone marrow samples (divided into two groups: tall-legged group with 8 samples and short-legged group with 7 samples) according to the manufacturer’s instructions for TRIzol Reagent (Life Technologies, California, USA). RNA concentration and purity were measured using a NanoDrop 2000 (Thermo Fisher Scientific, Wilmington, DE, USA). RNA integrity was assessed with the RNA Nano 6000 Kit on an Agilent Bioanalyzer 2100 system (Agilent Technologies, CA, USA). After passing the quality inspection, 1 μg of total RNA from each sample was used for library construction. Sequencing of the libraries was performed on the Illumina NovaSeq platform by Beijing Baimake Biotechnology Co., Ltd. The resulting clean reads were aligned to the chicken reference genome (Gallus_gallus.GRCg7b.genome.fa) using Hisat2 (2.0.4) to obtain positional information of reads on the reference genome (16). The aligned reads were then assembled using StringTie (17) to reconstruct the transcriptome for subsequent analysis. Based on the positional information aligned to the genome, read counts for each transcript were calculated, and gene expression levels were quantified using FPKM values.

#### 2.3.2 Differential expression gene data enrichment analysis

##### 2.3.2.1 Selection of DEGs

DEGs were identified based on the count values across samples using differential analysis software. For groups with biological replicates, DESeq2 software was employed for differential analysis; for groups without biological replicates, edgeR software was used. The selection criteria for DEGs included a Fold Change (FC) ≥ 1.5 and a *p*-value <0.01. The Fold Change represents the ratio of expression levels between two samples (or groups). The False Discovery Rate (FDR) was calculated to assess the significance of the differences based on the adjusted *p*-values. To facilitate comparisons, the Fold Change values were log-transformed (log_2_FC), where a larger absolute value of log_2_FC and a smaller FDR indicate more pronounced differences between the two groups.

##### 2.3.2.2 GO enrichment analysis

The differential expression genes were functionally annotated using the GO database, and statistical classification was performed at the secondary classification level. For each group of DEGs, enrichment analyses for biological processes, molecular functions, and cellular components were conducted using the ClusterProfiler package with hypergeometric testing. The resulting GO nodes from the enrichment analyses were visualized, and a Directed Acyclic Graph (DAG) was generated using topGO.

##### 2.3.2.3 KEGG enrichment analysis

The KEGG annotation results for the DEGs were classified according to pathway types in KEGG. Through KEGG annotation analysis, metabolic pathways related to the target genes were identified, elucidating the sources of phenotypic differences from biochemical and metabolic perspectives. The enrichment results were visualized using ClusterProfiler.

##### 2.3.2.4 Protein–protein interaction network

The sequences of the differential genes were aligned to the genomes of relevant species, with their predicted protein–protein interaction (PPI) relationships obtained from the STRING database (18).^3^ The PPI networks of these differential genes were then visualized using Cytoscape (19).

### 2.4 Candidate gene screening

Intersection analysis was performed on the genes obtained from resequencing and transcriptome sequencing, combining data from the GeneCards database and relevant literature to clarify the functions of intersecting genes. KEGG enrichment analysis was conducted using DAVID (see text footnote 1), and a Venn diagram was created to visualize the enrichment results with the online plotting software WeiShengXin (see text footnote 2). A protein interaction network diagram for the intersecting genes was constructed using the STRING database. Candidate genes related to the tall and short traits of Baicheng Fatty chickens were identified.

## 3 Results

### 3.1 Identification of candidate genes for tall and short traits using whole genome resequencing

#### 3.1.1 Genome data description

After quality control and alignment, the BAM files of 25 samples from the Baicheng Fatty chickens breed were statistically analyzed using Qualimap software. The alignment rate of clean reads from all samples to the reference genome was 99.50%, with an average sequencing depth of 11.36× and an average Q30 score of 92.79%. A total of approximately 1014.00 Mb of clean reads was obtained, generating about 302.52 Gbp of clean data (Supplementary Table 1).

#### 3.1.2 Whole genome genetic variation detection

**Figure 1:**
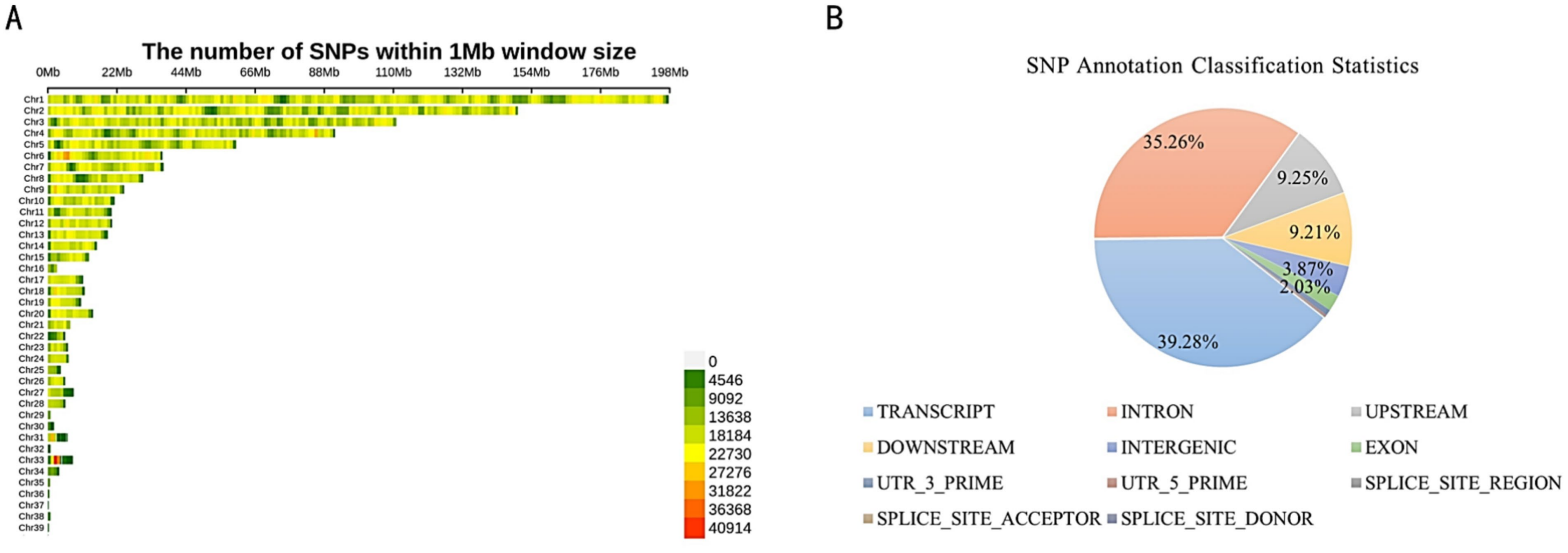
**(A)** Chromosomal distribution of SNPs. **(B)** SNP Annotation classification statistics.

A total of 28,897,168 SNP loci were identified through variation detection, with 5,518,574 SNPs remaining after filtering. Tall-legged chickens and short-legged chickens were found to have 14,335,980 and 14,561,188 SNP loci, respectively. The distribution of the 5,518,574 identified SNPs across the chromosomes indicated varying degrees of distribution on each chromosome (Figure 1A). The highest number of distributed SNPs was found on chromosome 1, followed by chromosome 2. Chromosomes 10, 11, 12, and 13 exhibited a similar distribution of SNPs. Annotation analysis revealed that SNPs are predominantly distributed across various genomic regions. The largest proportion, 39.28%, is found in the TRANSCRIPT region, followed closely by INTRON at 35.26%. The UPSTREAM and DOWNSTREAM regions account for 9.25 and 9.21%, respectively. The INTERGENIC region represents 3.87%, while SNPs in the EXON region make up 2.03%. Smaller proportions are found in the 3’ UTR (0.76%), 5’ UTR (0.20%), SPLICE_SITE_REGION (0.12%), SPLICE_SITE_ACCEPTOR (0.01%), and SPLICE_SITE_DONOR (0.01%) (Figure 1B).

#### 3.1.3 Population genetic structure analysis

##### 3.1.3.1 Principal component analysis (PCA) and linkage disequilibrium (LD)

**Figure 2:**
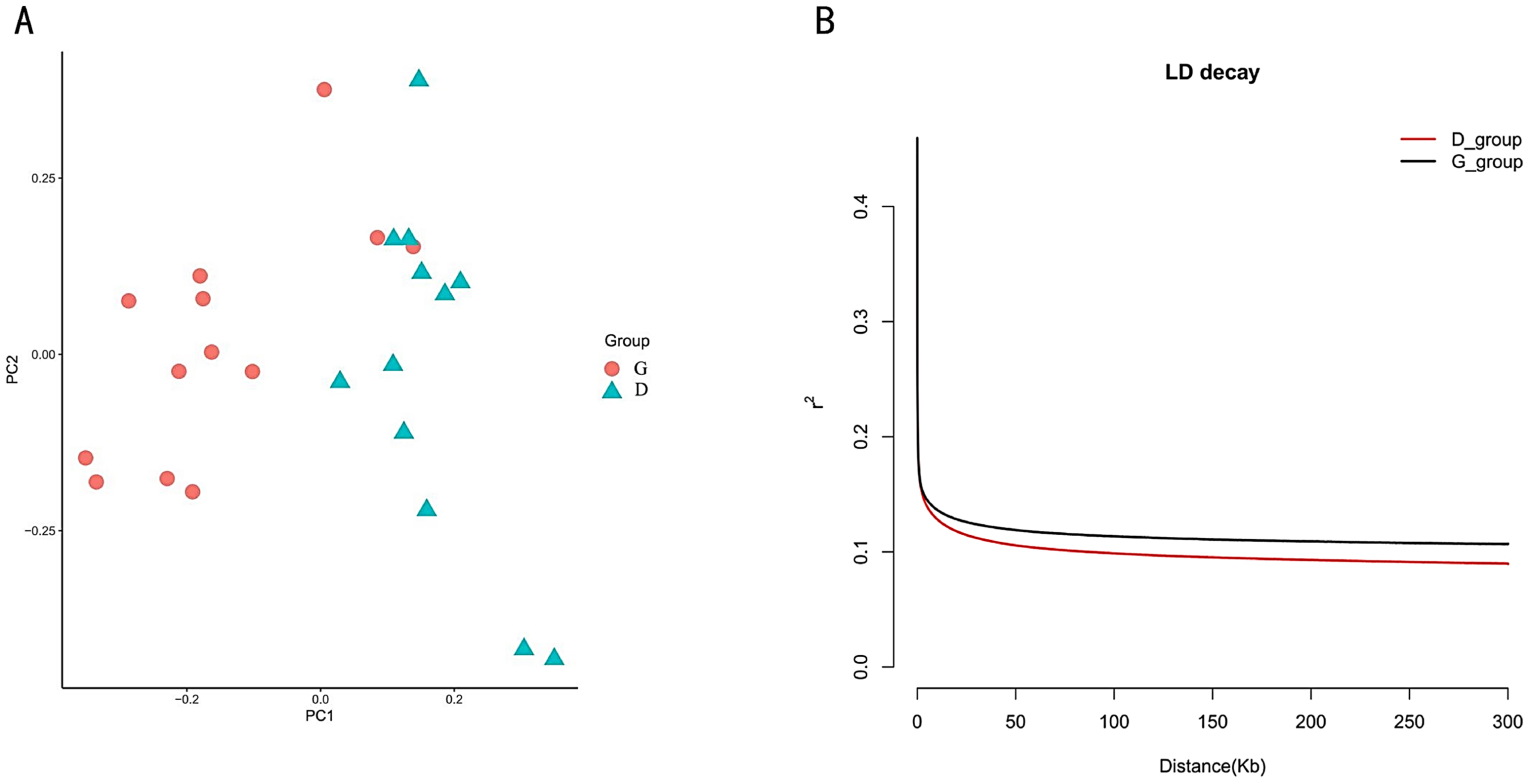
**(A)** PCA results: the orange circles represent the tall-legged chickens (G_group), and the green triangles represent the short-legged chickens (D_group). **(B)** LD analysis: the black curve represents the tall-legged chickens (G_group), and the red curve represents the short-legged chickens (D_group).

We conducted a population structure analysis, and the PCA results indicated that the tall-legged and short-legged groups could be clearly separated in terms of genetic structure (Figure 2A). For all SNPs, we calculated the LD, revealing that when the LD coefficient dropped to half of its maximum value, the tall-legged group (G_group) showed LD at 150 kb (*R*^2^ = 0.11), while the short-legged group (D_group) displayed a quicker decay at the same distance (*R*^2^ = 0.01) (Figure 2B).

##### 3.1.3.2 ROH analysis

**Figure 3:**
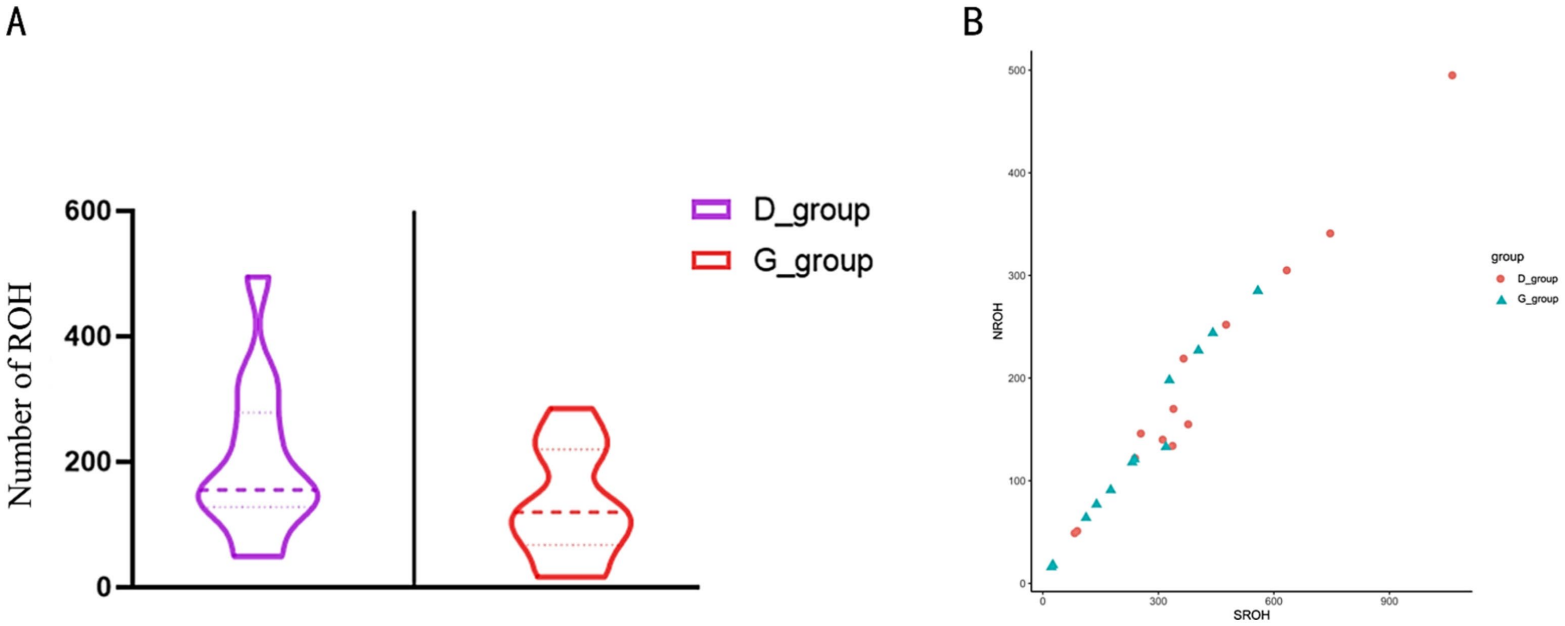
**(A)** Total number of ROH. The left side of the figure represents the short-legged chickens (D_group), and the right side represents the tall-legged chickens (G_group). **(B)** Relationship between NROH and SROH. The *y*-axis represents NROH, ranging from 0 to 500; the *x*-axis represents SROH, ranging from 0 to 900. The figure contains two sets of data, represented by different shapes and colors: red circles for the short-legged chickens (D_group) and blue triangles for the tall-legged chickens (G_group).

By comparing the number of individual ROH segments and their coefficients of variation in the tall and short-legged groups, we observed differences in genetic diversity and inbreeding levels between the two groups. Statistical analysis revealed that the short-legged group had an average of 198.4 ± 124.4 ROH segments, indicating significant variation in ROH numbers among individuals. Most individuals exhibited fluctuations around this average of 198.4, with a coefficient of variation of 62.73%. In contrast, the tall-legged group had an average of 132.7 ± 25.44 ROH segments, suggesting a certain degree of variation as well, but to a lesser extent; most individuals fluctuated around 132.7 with a coefficient of variation of 19.17% (Supplementary Table 2 and Figure 3A). Further analysis of the relationship between the number of homozygous segments (NROH) and the total length of homozygous segments (SROH) provided insights into the distribution characteristics of homozygous segments across different populations. We found that as NROH increased, data points for both the D_group and G_group showed a trend of increasing SROH, indicating a positive correlation between SROH and NROH (Figure 3B).

#### 3.1.4 Selection signal analysis

##### 3.1.4.1 Identification of selection signals using *F_ST_* and θ*π* ratios

**Figure 4:**
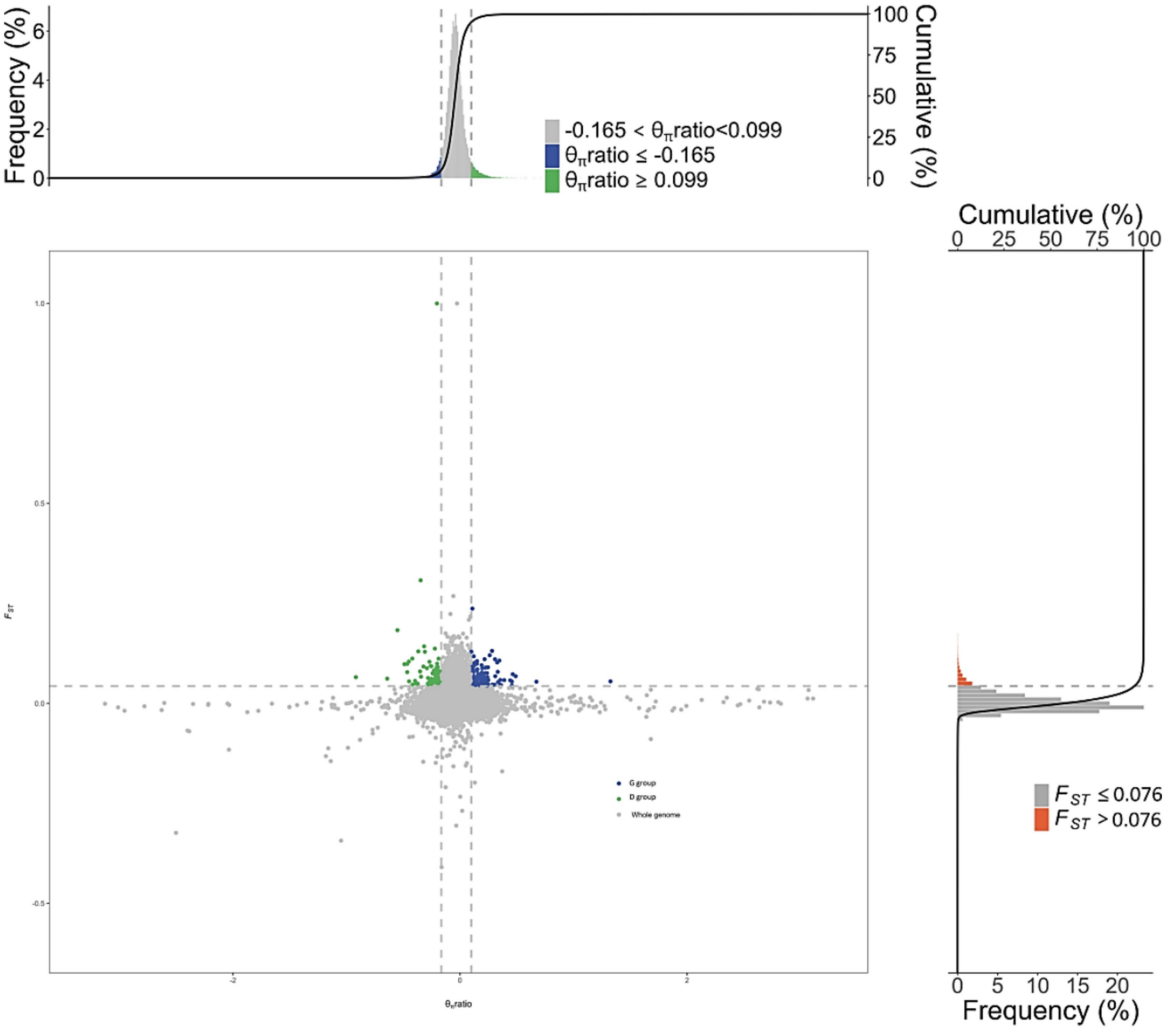
Visualization of selected gene regions in tall-legged and short-legged chickens.

Selection signal analysis was conducted using *F_ST_* and π ratio, with the intersection of the top 1% *F_ST_* (Supplementary Table 3) and the top 1% π ratio results identified as selected regions. This analysis revealed a total of 460 selected regions, which annotated 1,019 candidate genes (Figure 4).

##### 3.1.4.2 Annotation and functional enrichment of candidate genes

**Figure 5:**
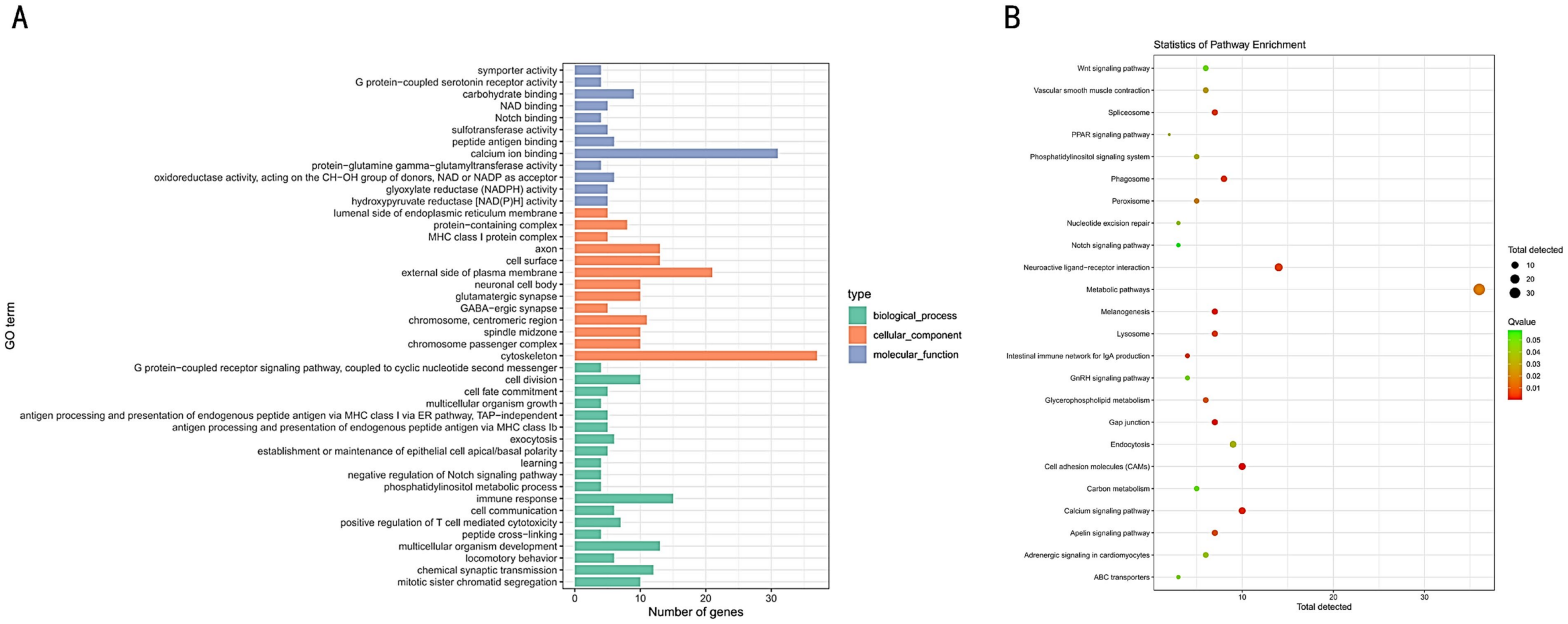
**(A)** GO functional enrichment analysis of selected genes in tall and short chickens. **(B)** KEGG pathway enrichment analysis of selected genes in tall and short chickens.

GO enrichment and KEGG pathway analyses of the selected regions in tall and short chickens were performed using DAVID and KOBAS websites. The GO enrichment analysis identified 44 GO terms, predominantly associated with processes such as Notch binding, G protein-coupled serotonin receptor activity, GABAergic synapse, and immune response (Figure 5A). The KEGG enrichment analysis identified a total of 24 pathways, primarily enriched in the WNT signaling pathway, PPAR signaling pathway, metabolic pathways, gap junction, and Notch signaling pathway (Figure 5B).

### 3.2 Identification of DEGs associated with tall and short traits using transcriptome sequencing

#### 3.2.1 Transcriptome data description

Transcriptome sequencing was performed on bone marrow tissue samples from 15 Baicheng Fatty chickens, including 8 tall-legged and 7 short-legged individuals. A total of 94.94 Gb of clean data was obtained, with each sample yielding at least 5.98 Gb of data. The Q30 base percentage was above 94.59%, and the GC content was consistently above 50.44%. Clean reads for each sample were aligned to the designated reference genome, with mapping efficiency ranging from 92.13 to 95.33%, indicating that the sequencing data met the requirements for subsequent analysis (Supplementary Table 4).

#### 3.2.2 Analysis of differentially expressed gene selection results

**Figure 6:**
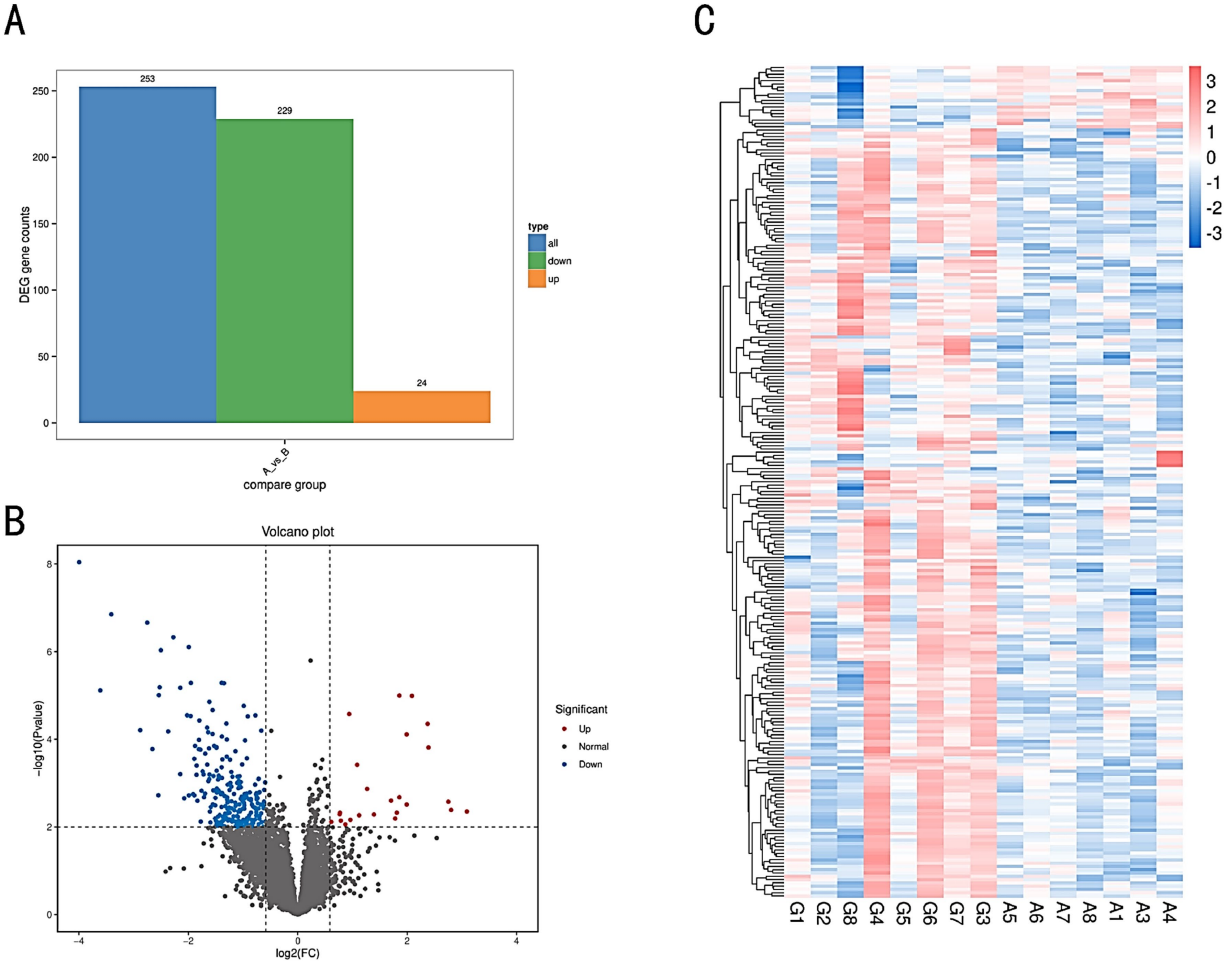
**(A)** Bar chart of differential gene statistics. The *x*-axis represents different sets of DEGs, with blue indicating all DEGs, orange representing upregulated genes, and green representing downregulated genes. The *y*-axis represents the number of DEGs. **(B)** Volcano plot of DEGs in comparison groups. **(C)** Heatmap of differentially expressed gene clustering. The *x*-axis represents the sample names and the clustering results of the samples, while the *y*-axis represents the differentially expressed genes and their clustering results. Each column corresponds to a different sample, and each row corresponds to a different gene. The color scale indicates the expression level of genes across the samples.

The number of DEGs in each comparison group is illustrated in a bar chart (Figure 6A), and the differential expression is visualized in a volcano plot (Figure 6B). A total of 253 DEGs were identified, with 229 genes upregulated and 24 genes downregulated. Genes exhibiting similar expression patterns may have analogous functions. A hierarchical clustering analysis was performed on all identified DEGs to cluster genes with the same or similar expression patterns across different samples, displayed through a heatmap (Figure 6C). This clustering allows for the identification of potentially related functions based on known functions of the clustered genes.

#### 3.2.3 GO and KEGG enrichment analysis of differential genes

**Figure 7:**
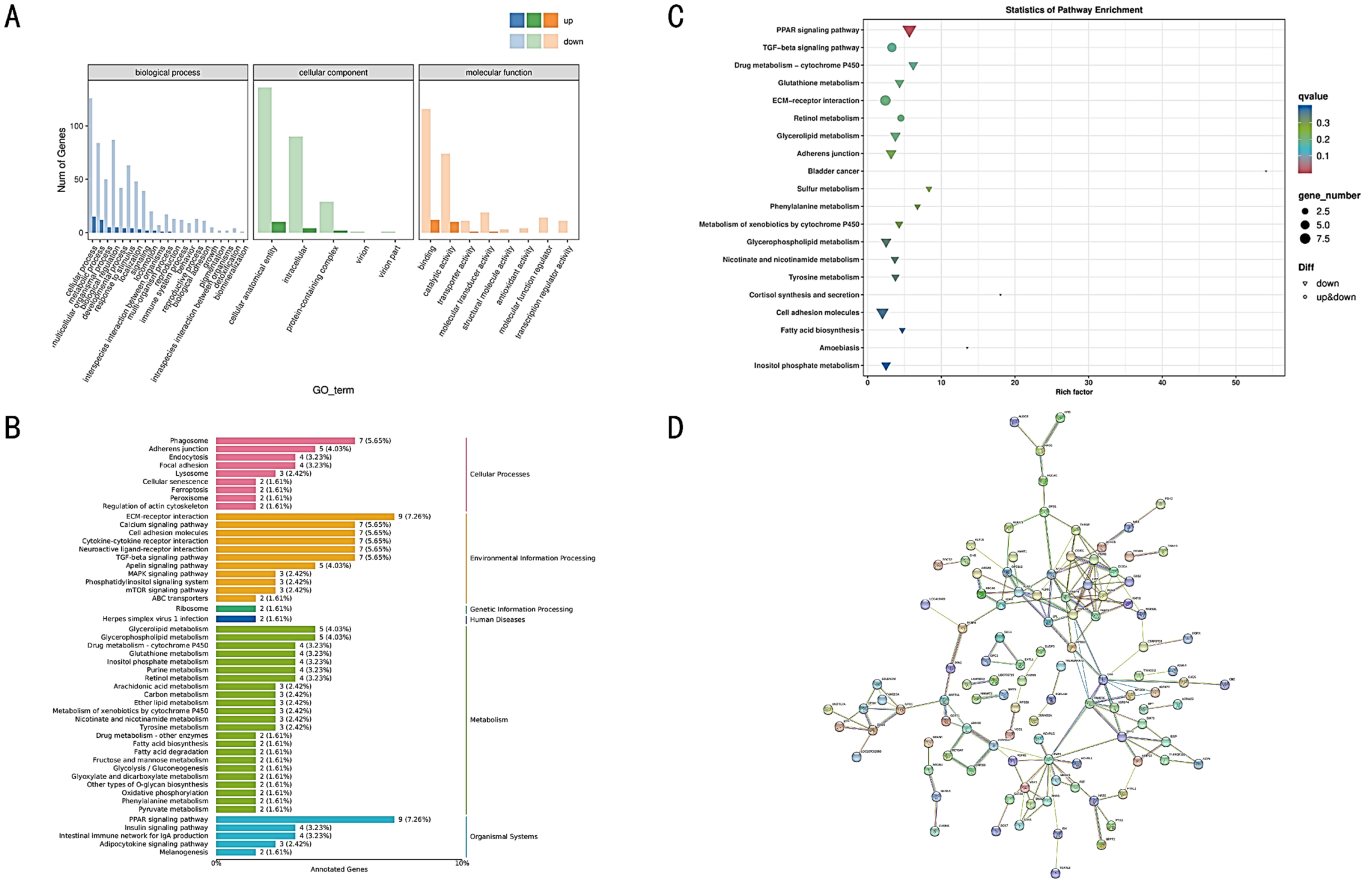
**(A)** GO annotation classification statistics of DEGs. **(B)** KEGG classification of DEGs. **(C)** KEGG enrichment bubble chart of DEGs. **(D)** Protein–protein interaction network of DEGs. Each circle represents a protein, with a simplified molecular structure diagram inside the circle, and the edges represent interaction relationships.

In this study, GO database was utilized to functionally annotate the DEGs, and statistical classification was performed at the secondary classification level. The GO annotation system encompasses three primary branches: Biological Process, Molecular Function, and Cellular Component. A total of 34 GO terms were identified in the enrichment analysis, primarily associated with Cellular Process, Metabolic Process, Developmental Process, Cellular Anatomical Entity, and Binding Activity (Figure 7A). To determine the GO terms significantly enriched compared to the overall genomic background, we employed the ClusterProfiler software to conduct enrichment analysis for Biological Processes, Molecular Functions, and Cellular Components using hypergeometric tests for each group of differential genes. The enrichment results were visualized to represent the GO nodes. The significance of functional pathways was assessed based on the q-value, where smaller *q*-values indicate greater significance. Using topGO, a Directed Acyclic Graph (DAG) was generated to intuitively display the enriched GO nodes of the DEGs and their hierarchical relationships. The branches in the DAG represent containment relationships, with the functional scope narrowing from top to bottom. The five most significantly enriched GO terms from each comparison group were selected as primary nodes, showcasing their associated GO terms (Supplementary Figure 1).

DEGs were annotated in KEGG, and the results were categorized according to the types of pathways within KEGG. Among these pathways, Metabolism represented the largest category (Figure 7B). The KEGG enrichment analysis revealed significant associations with pathways such as the TGF-beta signaling pathway, ECM-receptor interaction, Adherens junction, PPAR signaling pathway, and Glycerolipid metabolism (Figure 7C). Additionally, through KEGG annotation, metabolic pathways relevant to the target genes were identified from biochemical metabolic pathways, helping to elucidate the underlying causes of phenotypic differences (Supplementary Figure 2).

A protein–protein interaction (PPI) network for the DEGs was constructed using the STRING database. This database contains predicted and experimentally validated PPI data across multiple species, including both direct physical interactions and indirect functional associations. By aligning the DEGs with proteins in the database, homologous proteins were identified, and interaction pairs were established. The PPI network was visualized using Cytoscape software (Figure 7D), facilitating the prediction of interactions among genes and narrowing down the critical gene set, thus providing reliable candidates for subsequent functional studies.

### 3.3 Candidate gene selection

**Figure 8:**
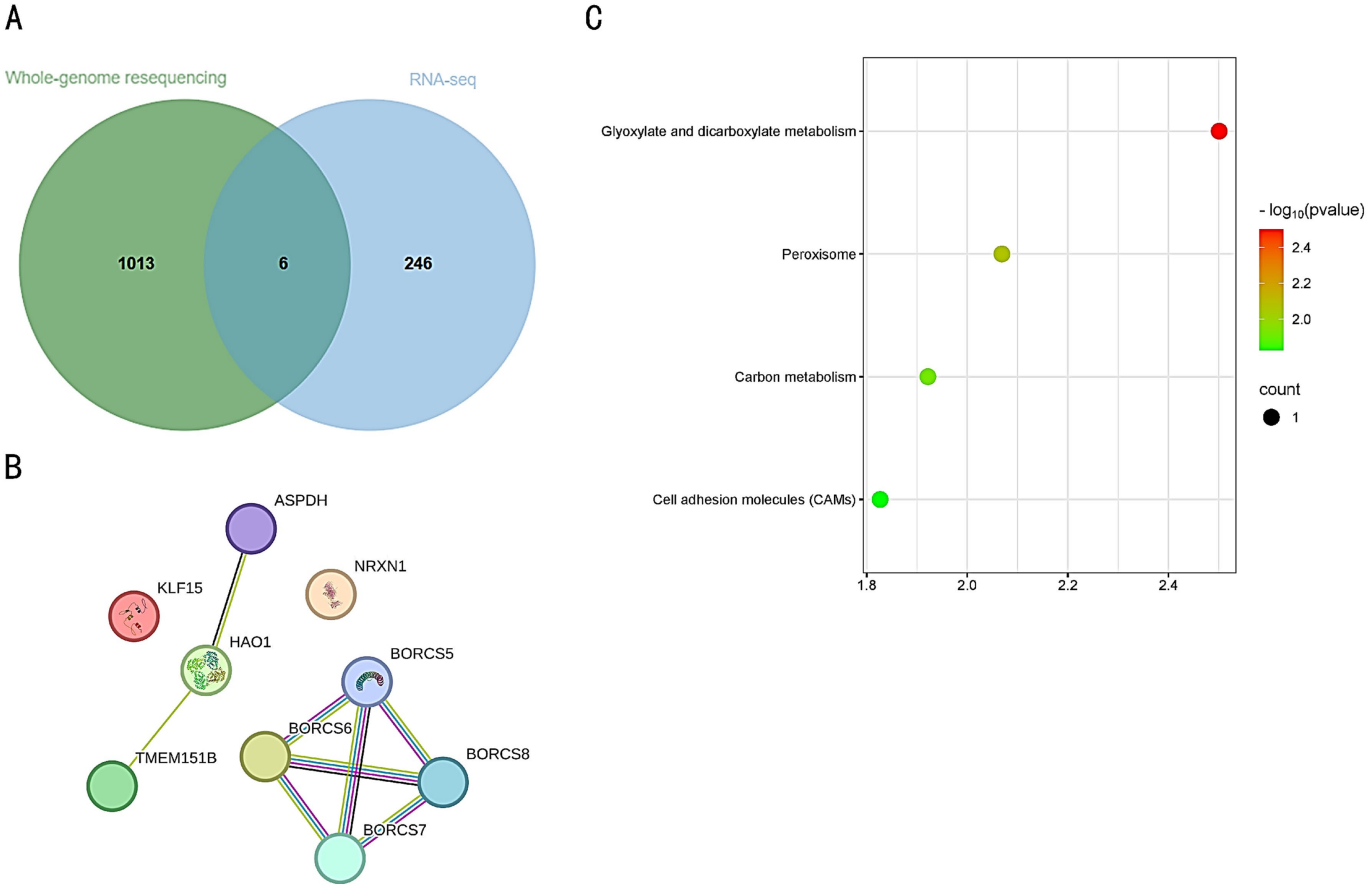
**(A)** Intersection of resequenced genes and DEGs. The green circle represents the resequenced genes, with a total of 1,013 genes. The blue circle represents the differentially expressed genes (DEGs), with a total of 247 genes. The overlapping area represents the intersection of the two, with a total of 6 genes. **(B)** Protein–protein interaction network of the intersected genes. Each circle represents a protein, with a simplified molecular structure diagram inside the circle, and the edges represent interaction relationships. **(C)** KEGG pathway enrichment analysis of the intersected genes. Each data point represents a pathway, with color and size indicating its significance level and the number of genes involved.

An intersection analysis was conducted between the genes identified from resequencing and the DEGs, resulting in the identification of six key genes: *LOC107050638*, *MHCY11*, *KLF15*, *HAO1*, *NRXN1*, and *BORCS6* (Figure 8A). A protein–protein interaction (PPI) network was constructed using the STRING database. Although the proteins displayed may not have direct interactions, this analytical approach aids in screening and identifying potential key genes, providing a foundation for further experimental validation (Figure 8B). KEGG analysis revealed that these candidate genes were primarily enriched in pathways related to Glyoxylate and dicarboxylate metabolism, the Peroxisome pathway, Carbon metabolism, and Cell adhesion molecules (CAMs) pathway (Figure 8C).

## 4 Discussion

The genomic and transcriptomic technologies are developing so fast that poultry genetic trait studies have deeply reached an unprecedented depth. In this study, we integrated whole-genome resequencing and transcriptomic sequencing data to investigate the genetic structural differences and molecular mechanisms underlying these two leg-length phenotypes (high-legged and short-legged) in Baicheng Fatty chickens.

### 4.1 Analysis of population genetic structure

In this study, two-dimensional PCA analysis revealed substantial genetic differences between the high-legged and short-legged groups. This finding is consistent with the results of Nie et al. (21), who identified strong genetic differentiation in chicken populations with different leg types through whole-genome resequencing. Wang et al. (22) demonstrated that domestication and selection acted on genomic regions associated with leg type, followed by the selection of regions under differential pressures, leading to genetic differentiation between leg type groups. Similar patterns of differentiation have been reported in other poultry breeds. For instance, Liu et al. (23) conducted a genomic-level population structure analysis, which revealed significant genetic differences between populations exhibiting distinct body size traits, as confirmed by phenotype analysis. Beijing oil chickens with different body size traits were identified, and a genome-wide association study (GWAS) was performed to pinpoint several candidate loci associated with weight and body size. These findings provide valuable insights into the genetic basis of body size traits in local chicken breeds.

LD analysis demonstrated that the decay rate in the short-legged group (*R*^2^ = 0.01) was significantly faster than that in the high-legged group (*R*^2^ = 0.11). Sheng et al. (24) studied hybrid populations between Chinese local chickens and commercial broilers, finding significant LD differences in genomic regions associated with growth traits among populations with different growth types. This difference is often a consequence of long-term selection acting on the genomic architecture, which aligns with the faster LD decay observed in the short-legged group. Their findings suggest that changes in LD patterns are associated with selective constraints on growth-related genes, providing important insights into the genetic basis of the high-legged and short-legged phenotypes. Rubin et al. (25) showed that different intensities of artificial selection on genomic regions during domestication led to distinct LD patterns, with LD decaying more rapidly near regions under strong selection. This supports the hypothesis that short-legged individuals may have experienced stronger selection pressure, resulting in more rapid LD decay. Additionally, their research elucidates how these selection patterns influence genome evolution, offering theoretical support for the mechanisms responsible for the creation of different phenotypic traits in chickens.

ROH analysis revealed variation in population genetic diversity. The short-legged group exhibited a higher number of ROH (198.4 ± 124.4) and a greater coefficient of variance (62.73%). Studies by Peripoll et al. (26) highlighted that increased levels of ROH often indicate selective pressure or bottleneck effects within a population. According to their research, the length distribution and number of ROH can reflect a population’s genetic history, and greater variation in the number of ROH suggests more intense artificial selection. This is consistent with the elevated ROH number (198.4 ± 124.4) and coefficient of variation (62.73%) observed in the short-legged group, suggesting stronger selection events in this group compared to the high-legged group.

### 4.2 Key genes and the analysis of their regulatory network

Through integrated analysis, this study identified six potential candidate genes, among which KLF15, HAO1, and NRXN1 are particularly well-studied. KLF family transcription factors play a crucial role in skeletal muscle development. Zhang et al. (27) demonstrated that the KLF family member Siha promotes skeletal muscle atrophy via the classical Wnt/*β*-catenin signaling pathway in chickens, suggesting that other KLF family members may also be pivotal in muscle development. KLF15, a member of the KLF family, regulates skeletal muscle development by associating with PPARδ to control lipid metabolism, which is essential for normal skeletal muscle function (28). Jung et al. (29) reported that KLF15 is a key molecule linking ER stress to metabolic regulation, influencing tissue growth and development by mediating amino acid and lipid metabolism. Their findings provide a molecular mechanism for understanding the role of KLF15 in myogenesis. Additionally, recent studies have shown that KLF15 is involved in regulating the growth plate of bones (30).

The primary impact of the HAO1 gene on skeletal development appears to be through its involvement in regulating the tricarboxylic acid cycle. Kimura et al. (31) used an HAO1-deficient mouse model to reveal the significant role of this gene in energy metabolism. Johnsson et al. (32) found a strong correlation between skeletal growth rate and HAO1 gene expression. These differences in energy metabolism may contribute to the phenotypic variations between the two sexes of chickens, specifically in those with extreme obesity, high-leg, and short-leg traits.

The potential role of NRXN1 in skeletal development has been more recently discovered. Gong et al. (33) reported that PRC2 modulates chondrocyte differentiation, while Wang et al. (34) demonstrated that NRXN1 regulates bone synthesis through calcium signaling pathways. These studies uncover the molecular mechanisms behind the high-legged and short-legged phenotypes.

### 4.3 Signaling pathways and their regulation of cell fate determination

KEGG analysis showed that the significant genes identified were predominantly enriched in metabolic-related pathways. Zhang et al. (35) found that the Glyoxylate and Dicarboxylate Metabolism pathway is strongly linked to skeletal growth, regulating energy metabolism to influence the proliferation and differentiation of growth plate chondrocytes. Buchert et al. (36) showed that the Cell Adhesion Molecules (CAMs) pathway is critical in regulating skeletal development through the interaction between chondrocytes and the extracellular matrix.

The Peroxisome pathway, recently studied for its significance in skeletal development, was also enriched in our analysis. Wang et al. (37) demonstrated that this pathway affects bone growth by regulating lipid metabolism and oxidative stress response. Yin et al. (38) further provided evidence that disrupting the Peroxisome pathway leads to skeletal development defects. These findings correspond to the observed variations in metabolic pathways between the high-legged and short-legged chickens in this study.

### 4.4 Importance and potential applications of the research

The results of this study have both theoretical significance and practical value for poultry breeding. By identifying key genes and pathways associated with leg-type traits, this study provides insights into the molecular mechanisms underlying poultry skeletal development. It also enhances the understanding of the relationships between these traits when compared with previous QTL-mapping studies, such as those by Wang et al. (37), which are essential for optimizing poultry breeding strategies. Selection breeding based on functional genomics has been shown to improve breeding efficiency significantly (39).

Furthermore, the candidate genes identified in this study can be applied in molecular marker-assisted selection (MAS). As demonstrated by Moniruzzaman et al. (40), MAS not only increases breeding efficiency but also improves selection accuracy, which is crucial for the protection and development of local chicken varieties, such as Baicheng oil chicken. Prior studies (41) have shown that combining genomic and transcriptomic data allows for more accurate breeding value predictions.

This research methodology and analytical framework can be applied to other poultry breeds, offering an integrative approach to identifying the genetic underpinnings of complex traits (42, 43). Future studies should validate these candidate genes to provide more reliable molecular tools for poultry breeding. Additionally, functional validation experiments are needed to confirm the specific roles of these genes in the development of leg-type traits in Baicheng oil chickens, and gene editing approaches could be employed in future studies to further elucidate their functions.

## 5 Conclusion

This study identified a total of 28,897,168 SNP loci through whole-genome resequencing and uncovered 253 DEGs using transcriptome sequencing. Combined analysis revealed six key genes: *KLF15*, *LOC107050638*, *MHCY11*, *HAO1*, *NRXN1*, and *BORCS6*. Among these, *KLF15* is highlighted as a critical gene associated with growth traits, potentially playing an important role in the regulation of skeletal muscle growth in chickens.

## Data Availability

The datasets generated or analyzed in this study are available in the National Genome Data Centre of the Beijing Institute for Genome Research, Chinese Academy of Sciences (National Center for Biological Information, China) under accession number PRJCA035911.

## Footnotes

^1^ https://david.ncifcrf.gov/

^2^ http://www.bioinformatics.com.cn/

^3^ http://string-db.org/
