# Supplementary material for: Integration of whole genome resequencing and transcriptome sequencing to identify candidate genes for tall and short traits in Baicheng Fatty chickens
Source: Front Vet Sci. 2025 Feb 27;12:1534742. doi: 10.3389/fvets.2025.1534742 (PMC11903400; doi:10.3389/fvets.2025.1534742)
Supplement: Supplementary file 1 [file Data_Sheet_1.pdf]

Supplementary Figure:

$P_{2\text{-}4}$: TopGO-directed acyclic graph of differentially expressed genes

P_5: Illustration of KEGG pathway annotation for differentially expressed genes

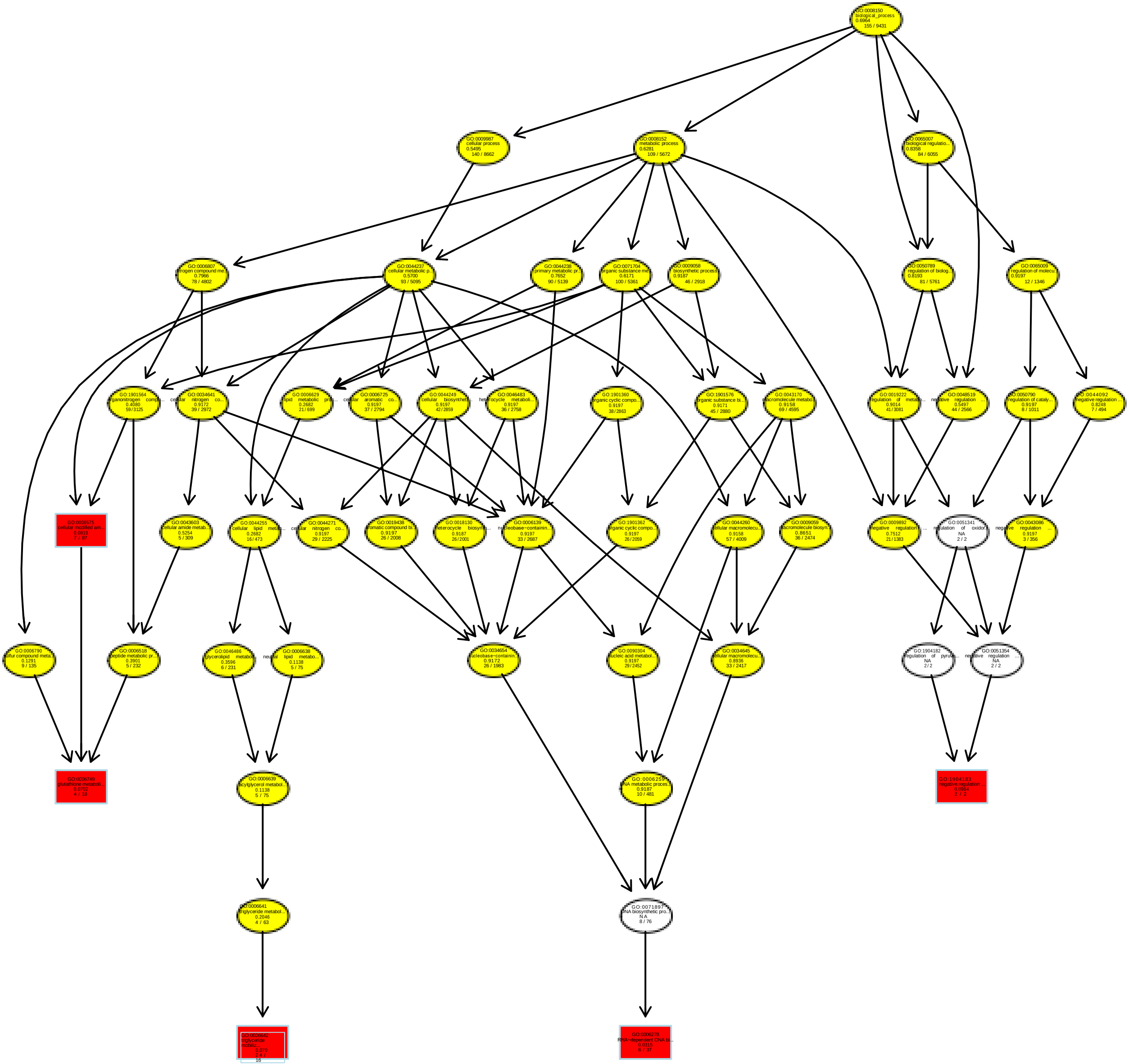
GO:0008150
biological_process
0.6964
155 / 9431
GO:0009987
cellular process
0.5495
140 / 8662
GO:0008152
metabolic process
0.6281
109 / 5672
GO:0065007
biological regulatio...
0.8358
84 / 6055
GO:0006807
nitrogen compound me...
0.7966
78 / 4802
GO:0044237
cellular metabolic p...
0.5700
93 / 5095
GO:0044238
primary metabolic pr...
0.7652
90 / 5139
GO:0071704
organic substance me...
0.6171
100 / 5361
GO:0009058
biosynthetic process
0.9187
46 / 2918
GO:0050789
regulation of biolog...
0.8193
81 / 5761
GO:0065009
regulation of molecu...
0.9197
12 / 1346
GO:1901564
organonitrogen compo...
0.4080
59 / 3125
GO:0034641
cellular nitrogen co...
0.9172
39 / 2972
GO:0006629
lipid metabolic proc...
0.2682
21 / 699
GO:0006725
cellular aromatic co...
0.9197
37 / 2794
GO:0044249
cellular biosyntheti...
0.9197
42 / 2859
GO:0046483
heterocycle metaboli...
0.9197
36 / 2758
GO:1901360
organic cyclic compo...
0.9197
38 / 2863
GO:1901576
organic substance bi...
0.9171
45 / 2880
GO:0043170
macromolecule metabo...
0.9158
69 / 4595
GO:0019222
regulation of metabo...
0.9014
41 / 3081
GO:0048519
negative regulation ...
0.5497
44 / 2566
GO:0050790
regulation of cataly...
0.9197
8 / 1011
GO:0044092
negative regulation ...
0.8248
7 / 494
GO:0006575
cellular modified am...
0.0819
7 / 87
GO:0043603
cellular amide metab...
0.5254
5 / 309
GO:0044255
cellular lipid metab...
0.2682
16 / 473
GO:0044271
cellular nitrogen co...
0.9197
29 / 2225
GO:0019438
aromatic compound bi...
0.9197
26 / 2008
GO:0018130
heterocycle biosynth...
0.9187
26 / 2001
GO:0006139
nucleobase-containin...
0.9197
33 / 2687
GO:1901362
organic cyclic compo...
0.9197
26 / 2059
GO:0044260
cellular macromolecu...
0.9158
57 / 4009
GO:0009059
macromolecule biosyn...
0.8651
36 / 2474
GO:0009892
negative regulation ...
0.7512
21 / 1383
GO:0051341
regulation of oxidor...
NA
2 / 2
GO:0043086
negative regulation ...
0.9197
3 / 356
GO:0006790
sulfur compound meta...
0.1291
9 / 135
GO:0006518
peptide metabolic pr...
0.3901
5 / 232
GO:0046486
glycerolipid metabol...
0.3596
6 / 231
GO:0006638
neutral lipid metabo...
0.1138
5 / 75
GO:0034654
nucleobase-containin...
0.9172
26 / 1983
GO:0090304
nucleic acid metabol...
0.9197
29 / 2452
GO:0034645
cellular macromolecu...
0.8936
33 / 2417
GO:1904182
regulation of pyruva...
NA
2 / 2
GO:0051354
negative regulation ...
NA
2 / 2
GO:0006749
glutathione metaboli...
0.0702
4 / 18
GO:0006639
acylglycerol metabol...
0.1138
5 / 75
GO:0006259
DNA metabolic proces...
0.9187
10 / 481
GO:1904183
negative regulation ...
0.0964
2 / 2
GO:0006641
triglyceride metabol...
0.2046
4 / 63
GO:0071897
DNA biosynthetic pro...
NA
8 / 76
GO:0006642
triglyceride mobiliz...
0.070
4 / 16
GO:0006278
RNA-dependent DNA bi...
0.0315
5 / 37

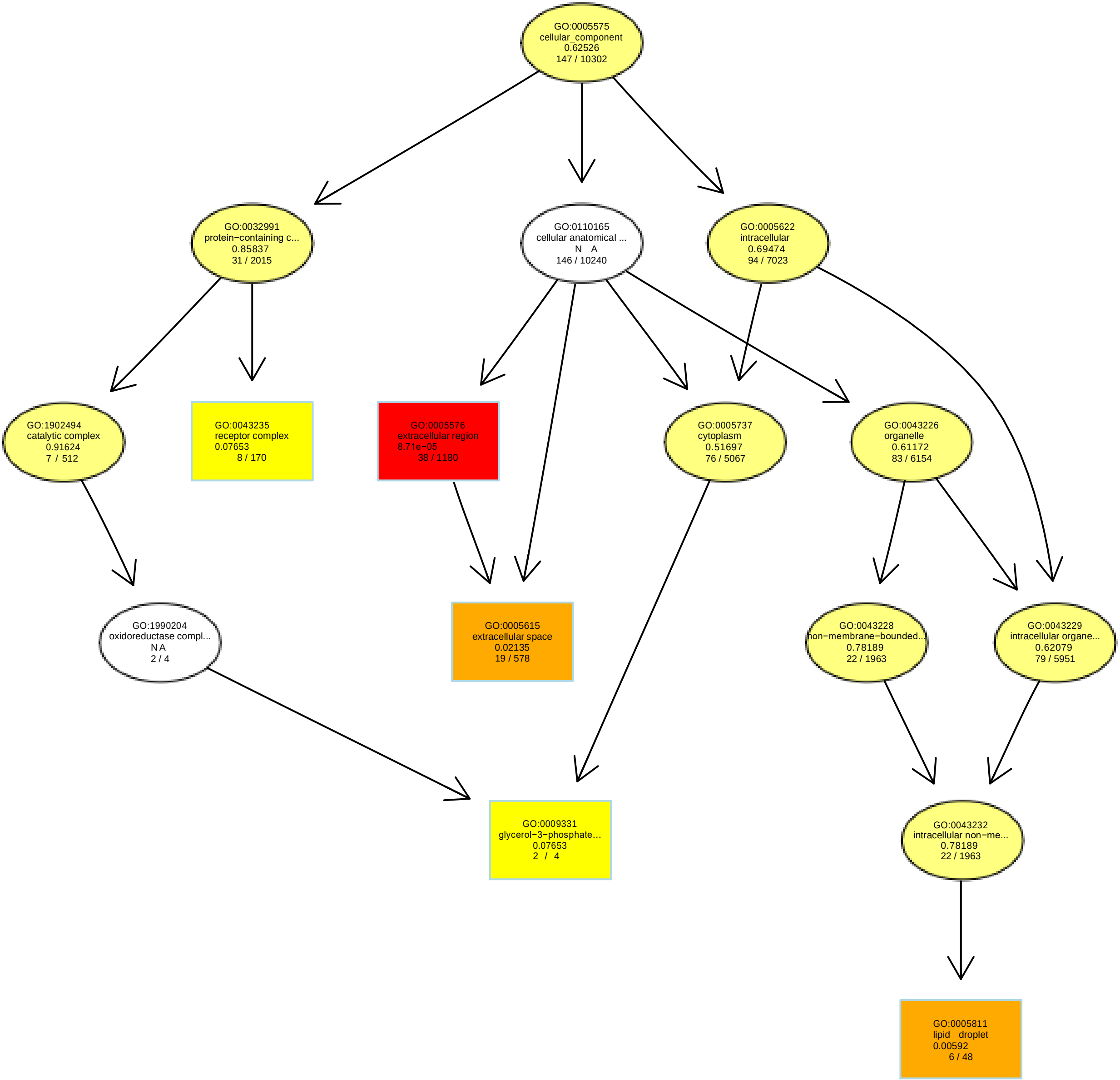
GO:0005575
cellular_component
0.62526
147 / 10302
GO:0032991
protein−containing c...
0.85837
31 / 2015
GO:0110165
cellular anatomical ...
N A
146 / 10240
GO:0005622
intracellular
0.69474
94 / 7023
GO:1902494
catalytic complex
0.91624
7 / 512
GO:0043235
receptor complex
0.07653
8 / 170
GO:0005576
extracellular region
8.71e−05
38 / 1180
GO:0005737
cytoplasm
0.51697
76 / 5067
GO:0043226
organelle
0.61172
83 / 6154
GO:1990204
oxidoreductase compl...
N A
2 / 4
GO:0005615
extracellular space
0.02135
19 / 578
GO:0043228
non−membrane−bounded...
0.78189
22 / 1963
GO:0043229
intracellular organe...
0.62079
79 / 5951
GO:0009331
glycerol−3−phosphate...
0.07653
2 / 4
GO:0043232
intracellular non−me...
0.78189
22 / 1963
GO:0005811
lipid droplet
0.00592
6 / 48

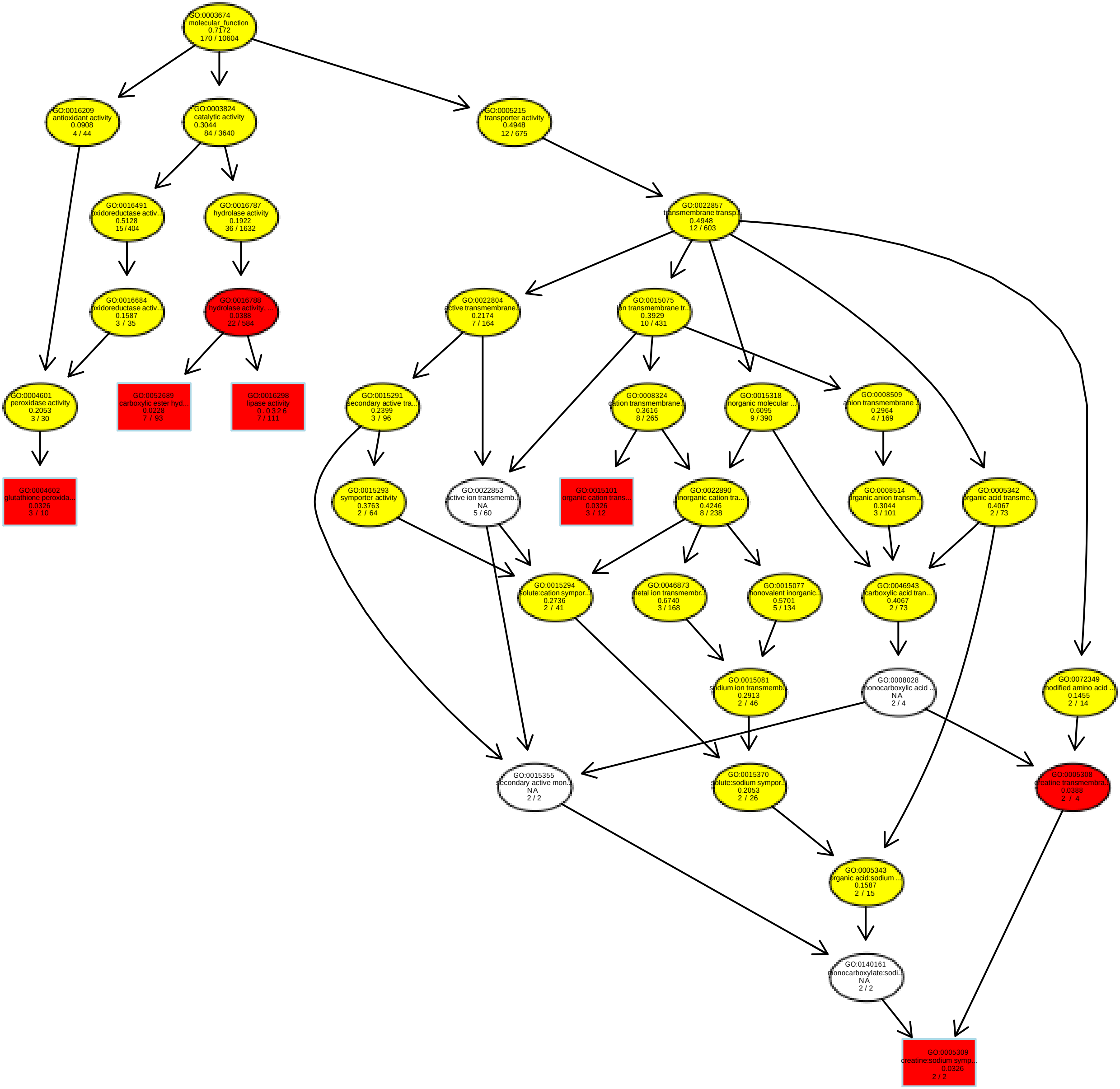

GO:0003674 molecular_function 0.7172 170 / 10604
GO:0016209 antioxidant activity 0.0908 4 / 44
GO:0003824 catalytic activity 0.3044 84 / 3640
GO:0005215 transporter activity 0.4948 12 / 675
GO:0016491 oxidoreductase activ... 0.5128 15 / 404
GO:0016787 hydrolase activity 0.1922 36 / 1632
GO:0022857 transmembrane transp... 0.4948 12 / 603
GO:0016684 oxidoreductase activ... 0.1587 3 / 35
GO:0016788 hydrolase activity, ... 0.0388 22 / 584
GO:0022804 active transmembrane... 0.2174 7 / 164
GO:0015075 ion transmembrane tr... 0.3929 10 / 431
GO:0004601 peroxidase activity 0.2053 3 / 30
GO:0052689 carboxylic ester hyd... 0.0228 7 / 93
GO:0016298 lipase activity 0.0326 7 / 111
GO:0015291 secondary active tra... 0.2399 3 / 96
GO:0008324 cation transmembrane... 0.3616 8 / 265
GO:0015318 inorganic molecular ... 0.6095 9 / 390
GO:0008509 anion transmembrane ... 0.2964 4 / 169
GO:0004602 glutathione peroxida... 0.0326 3 / 10
GO:0015293 symporter activity 0.3763 2 / 64
GO:0022853 active ion transmemb... NA 5 / 60
GO:0015101 organic cation trans... 0.0326 3 / 12
GO:0022890 inorganic cation tra... 0.4246 8 / 238
GO:0008514 organic anion transm... 0.3044 3 / 101
GO:0005342 organic acid transme... 0.4067 2 / 73
GO:0015294 solute:cation sympor... 0.2736 2 / 41
GO:0046873 metal ion transmembr... 0.6740 3 / 168
GO:0015077 monovalent inorganic... 0.5701 5 / 134
GO:0046943 carboxylic acid tran... 0.4067 2 / 73
GO:0015081 sodium ion transmemb... 0.2913 2 / 46
GO:0008028 monocarboxylic acid ... NA 2 / 4
GO:0072349 modified amino acid ... 0.1455 2 / 14
GO:0015355 secondary active mon... NA 2 / 2
GO:0015370 solute:sodium sympor... 0.2053 2 / 26
GO:0005308 creatine transmembra... 0.0388 2 / 4
GO:0005343 organic acid:sodium ... 0.1587 2 / 15
GO:0140161 monocarboxylate:sodi... NA 2 / 2
GO:0005309 creatine:sodium symp... 0.0326 2 / 2

OXIDATIVE PHOSPHORYLATION

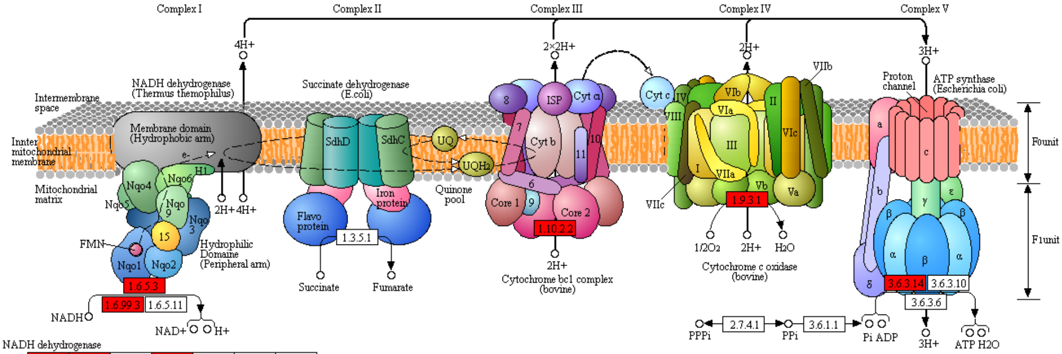

NADH dehydrogenase

E	ND1	ND2	ND3	ND4	ND4L	ND5	ND6										
E	Ndufs1	Ndufs2	Ndufs3	Ndufs4	Ndufs5	Ndufs6	Ndufs7	Ndufs8	Ndufv1	Ndufv2	Ndufv3						
B/A	NuoA	NuoB	NuoC	NuoD	NuoE	NuoF	NuoG	NuoH	NuoI	NuoJ	NuoK	NuoL	NuoM	NuoN			
B/A	NdhC	NdhK	NdhJ	NdhH	NdhA	NdhI	NdhG	NdhE	NdhF	NdhD	NdhB	NdhL	NdhM	NdhN	HoxE	HoxF	HoxU
E	Ndufa1	Ndufa2	Ndufa3	Ndufa4	Ndufa5	Ndufa6	Ndufa7	Ndufa8	Ndufa9	Ndufa10	Ndufab1	Ndufa11	Ndufa12	Ndufa13			
E	Ndufb1	Ndufb2	Ndufb3	Ndufb4	Ndufb5	Ndufb6	Ndufb7	Ndufb8	Ndufb9	Ndufb10	Ndufb11	Ndufc1	Ndufc2				

Succinate dehydrogenase / Fumarate reductase

E	SDHC	SDHD	SDHA	SDHB	
B/A	SdhC	SdhD	SdhA	SdhB	
			FrdA	FrdB	FrdC	FrdD

Cytochrome c reductase

E/B/A	ISP	Cyt b	Cyt 1				
E	COR1	QCR2	QCR6	QCR7	QCR8	QCR9	QCR10

Cytochrome c oxidase

E	COX10		COX3	COX1	COX2	COX4	COX5A	COX5B	COX6A	COX6B	COX6C	COX7A	COX7B	COX7C	COX8	
E/B/A	COX11	COX15	COX17													
B/A	CyoE	CyoD	CyoC	CyoB	CyoA											
		CoxD	CoxC	CoxA	CoxB											
		QoxD	QoxC	QoxB	QoxA											

Cytochrome c oxidase , cbb3-type

B	I	II	IV	III

Cytochrome bd complex

B/A	CydA	CydB

F-type ATPase (Bacteria)

alpha	beta	gamma	delta	epsilon
a	b	c		

F-type ATPase (Eukaryotes)

alpha	beta	gamma	delta	epsilon	
OSCP	a	b	c	d	e
f	g	f6/h	j	k	8

V/A-type ATPase (Bacteria, Archaeas)

A	B	C	D	E	F	G/H
I	K					

V-type ATPase (Eukaryotes)

A	B	C	D	E	F	G	H
a	c	d	e	S1			

00190 6/16/15
(c) Kanehisa Laboratories
